# Metalworking fluids and cancer mortality in a US autoworker cohort (1941–2015)

**DOI:** 10.5271/sjweh.3898

**Published:** 2020-09-01

**Authors:** Sadie Costello, Kevin Chen, Sally Picciotto, Liza Lutzker, Ellen Eisen

**Affiliations:** 1 Environmental Health Science, School of Public Health, University of California, Berkeley CA, USA

**Keywords:** auto manufacturing, cohort analysis, cohort study, Cox model, exposure, standardized mortality ratio

## Abstract

**Objectives:**

This report describes the extended follow-up (1941–2015) of a cohort of 38 549 automobile manufacturing workers with potential exposure to metalworking fluids (MWF). The outcomes of interest were mortality from cancers of the esophagus, stomach, intestine, rectum, bladder, liver, pancreas, larynx, lung, skin, prostate, brain, and female breast, as well as leukemia. This report includes 5472 deaths from cancer, more than ten times the numbers of deaths in our last summary report published 20 years ago.

**Methods:**

Standardized mortality ratios were computed for the entire study period. Adjusted hazard ratios (HR) were estimated in Cox proportional hazard models with categorical variables for cumulative exposure to each type of MWF.

**Results:**

Exposure–response patterns are consistent with prior mortality reports from this cohort. We found increased risk of skin and female breast cancer with straight fluids. For the first time, we found elevated risk of stomach cancer mortality. Overall, many of the exposure–response results did not suggest an association with MWF.

**Conclusions:**

Mortality is a poor proxy for cancer diagnosis for treatable cancers and not the optimal outcome measure in etiological studies. Although the HR presented here handle bias from the healthy worker hire effect and left truncation, they do not handle bias from healthy worker survivor effect, which likely results in underestimates of the health impacts of MWF. Although this updated summary provides some information on the risk of cancer from MWF, targeted future analyses will help clarify associations.

Metalworking fluids (MWF) are complex mixtures of oils and chemical additives widely used to cool and lubricate metal machining operations. MWF are aerosolized when sprayed, generating airborne particulate matter (PM) at concentrations up to two orders of magnitude higher than allowable by the US ambient air pollution standards (1). Classified as straight (mineral oils), soluble (oils emulsified in water), or synthetic (without oils), MWF continue to pose a potential hazard to millions of workers in automobile manufacturing as well as other metal machining jobs related to electronics manufacturing, new technologies, and alternative energy. Some MWF constituents are carcinogenic in animals, including N-nitrosamines (2) found in water-based synthetic fluids and some polycyclic aromatic hydrocarbons (PAH) (3) found in the oil-based fluids. Efforts to reduce exposures to these potentially carcinogenic MWF have been ongoing for decades. Removal of PAH from MWF began in the 1950s when large industrial users began shifting to more refined oils, and US Environmental Protection Agency (EPA) regulations during the 1980s were directed at reducing nitrosamine exposures (4). In 1998, the National Institute of Occupational Safety and Health (NIOSH) released a criteria document with a recommended exposure limit (REL) for occupational exposure to MWF of 0.5 mg/m^3^ for total PM (TPM) and 0.4 for respirable PM (5).

Several reviews of the evidence on MWF and cancer followed the NIOSH Criteria Document (4, 6–9). Calvert et al (4) summarized the evidence basis of the NIOSH report on cancer risk among workers exposed to MWF, concluding that there was substantial evidence for increased risk at several sites, including larynx, rectum, pancreas, skin, scrotum, and bladder, associated with at least some MWF. Savitz (6) concluded that evidence was strongest for associations between cancers of the larynx and rectum in relation to the oil-based fluids. Mirer (7, 8) noted positive results for stomach cancer in older studies and internal analyses of labor union [United Autoworkers (UAW)] data without quantitative exposure information, as well as for lung, liver, pancreatic, and laryngeal cancer, and for leukemia. In all reviews, attention was focused on air and skin exposure to the oil-based MWF in use before the oils became more highly refined in the mid-1970s. Most of the quantitative evidence cited in all the reviews came from the ongoing UAW-General Motors (GM) cohort study (10).

The UAW-GM study was jointly funded by labor and management as a cancer mortality study with an extensive exposure assessment component, motivated by worker concerns about digestive and respiratory cancers in relation to MWF exposure. Standardized mortality ratios (SMR) have been reported twice for this cohort, the first based on the original end of follow-up in 1985 and the second based on extended follow-up to 1995 (10, 11). SMR for the two outcomes of original interest, stomach and lung cancer, were not elevated in either report. A series of results from exposure–response analyses have also been reported based on the extensive historical exposure assessment for straight, soluble and synthetic MWF. Results based on Cox proportional hazard models for digestive and respiratory cancer mortality in relation to MWF exposures have been largely null (11–15). However, results based on cancer incidence in this cohort have been more mixed. There is modest evidence that exposure to straight, oil-based MWF increases the risk of laryngeal (14, 16), bladder (17), melanoma (18), breast (19), and colon (20), cancer incidence. Limited evidence was also reported for increased risk of cervical cancer (21) and breast cancer in younger women (19).

In 2003, the UAW petitioned the Occupational Safety and Health Administration (OSHA) for a temporary standard for MWF with an exposure limit of 0.5 mg/m^3^ TPM. The petition was based on the evidence for nonmalignant respiratory health effects of MWF, asthma and hypersensitivity pneumonitis, rather than for cancer. OSHA ultimately denied the petition (6). The UAW’s decision to petition for regulatory efforts based on nonmalignant health effects suggests that in 2003, there was insufficient evidence that MWF are carcinogenic at concentrations found in the workplace. Yet, a recent risk assessment for cancer and MWF based entirely on published results from the UAW-GM cohort study, concluded that substantial risk exists at 0.1 mg/m^3^ respirable PM, one quarter of the current NIOSH REL (and the internal GM limit) (9). The cancer sites contributing the most attributable cases were larynx, esophagus, brain, breast and cervix.

In summary, the literature to date suggests that oil- and water-based MWF may indeed cause increased risk of several specific cancers, although none of the evidence is conclusive. In this context, we report results from an extended vital status follow-up, from 1941 to 2015 – 20 years beyond the last reported mortality follow-up of the UAW-GM cohort.

## Methods

Details regarding the UAW-GM cohort mortality study have been described extensively in previous publications (10, 11, 22–24). Here, we describe the methods in brief.

### Study population

The present study of the UAW-GM cohort includes all hourly workers identified through company records at three automobile manufacturing plants in Michigan who worked for ≥3 years and were hired between 1 January 1938 and 31 December 1981. After excluding the 4% of subjects missing more than half of their employment history, 38 549 were included in this analysis. Follow-up for mortality now extends from 1941 to 2015, 21 years longer than the previous update (11) and includes more than 1.5 million person-years. Over the 74 years of follow-up, 53% of the study population has died. Subjects were considered lost to follow-up upon reaching the oldest observed age at death (106 years). By this definition, <0.5% of the participants were lost to follow-up.

### Covariates

Subject characteristics, including year of birth, sex (male or female), race (white, black, or unknown), and worksite (plant 1, 2, or 3) were obtained from company records. Subjects with unknown race (22%) were assumed to be white in this analysis based on available demographics (10). In a sensitivity analysis, subjects at plant 1 with missing race were assumed to be black.

### Exposure

Exposure assessment has been described in previous publications (23–25). Quantitative exposure to MWF was based on several hundred personal and area size-selective samples for PM (mg/m^3^) collected across jobs and departments by the research team, in combination with historical industrial hygiene records. Scale factors were applied to estimate historical levels of exposure relative to baseline measurements made by the research industrial hygienists (mid 1980s) (23). These scale factors reflect the dramatic decreases in exposure concentrations over the second half of the 20^th^ century, particularly in the early 1970s with the passage of the Occupational Safety and Health Act.

MWF exposures were assigned to individuals according to job and department and calendar time, weighted by work time. Missing exposure data were interpolated for those missing less than half of their work history. The exposure–response models considered exposure to straight, soluble, and synthetic MWF measured as cumulative exposure to TPM. The work history records were initially collected in 1985 and extended up to 1995. Exposure–response models for this analysis are based on cumulative MWF exposure (mg/m^3^-years) lagged by 21 years; lagging accounts for disease latency and is necessitated by the available data.

### Outcome

Data on vital status and cause of death were obtained through the Social Security Administration, the National Death Index, company records, death certificates, and state mortality files (10). Causes of death were selected for exposure–outcome modeling based on the previous report on cancer mortality in this cohort in 2001 (11).

### Analytic methods

Person-years were accumulated from three years after hire until death, end of follow-up, or the maximum observed age at death. Causes and dates of death were obtained from company records, the Social Security Administration, death certificates, state mortality files, and the National Death Index. Underlying causes of death were coded conforming to the International Classification of Diseases, revisions 9 and 10 [ICD-9 and ICD-10, respectively; see the supplementary material (www.sjweh.fi/show_abstract.php?abstract_id=3898) for ICD coding). Where possible, these ICD codes were mapped to cause of death descriptions according to the keys used in the Lifetable Analysis System (LTAS) (26–28). SMR were computed for cancer outcomes, as well as several chronic diseases and external causes of death. Reference rates for deaths prior to 2010 were extracted from LTAS; reference rates for deaths in or after 2010 were obtained through the CDC Underlying Cause of Death database (29, 30) and SMR were calculated using R version 3.6.1 (R Core Team, Vienna Austria).

We estimated associations between cumulative exposure to straight, soluble, and synthetic MWF and each cancer outcome as adjusted hazard ratios (HR) in Cox proportional hazards models with age as the timescale. In addition to age, all models included year of hire, race, sex, and plant, as well as time-varying calendar year and the other MWF exposures to adjust for potential confounding. Cumulative exposures to the three MWF were categorized with a pre-determined reference group. Zero exposure was the reference group for straight and synthetic fluid. For soluble exposures, a more ubiquitous exposure in this cohort, the upper bound of the reference group was set to 0.05 mg/m^3^ to avoid extremely small numbers of cancer cases in the reference group and thereby increase stability of the HR estimates. This cut-off is approximately 1% of what cumulative exposure would be after ten years at the NIOSH REL. To maximize statistical efficiency, we used the distribution of exposure to each fluid type among the cases of each cancer to determine the cut points for the exposed categories.

## Results

A summary of the study population characteristics is presented in table 1. Over half of this predominantly white and male cohort had died by the end of follow up in 2015. While at work, approximately half of the workers had been exposed to straight fluids, a third to synthetics, and a majority (82%) were exposed to soluble fluids. Although only a quarter of the workforce was employed at plant 1, most of the cohort members categorized as black worked at this urban plant (data not shown). Results are presented as SMR as well as adjusted HR, estimated in Cox models based on quantitative exposure estimates for each fluid type.

**Table 1:** Summary of study population characteristics (N=38 549; 1.51 million person-years). The cohort was restricted to individuals who were hired in or after 1938 and for whom at least half of their work history data was available. Individuals were considered lost to follow-up once they reached the maximum observed age at death.

	N	%	Median	Q1, Q3
Study population size	38 549	100		
Race				
White	22 816	59		
Black	7 131	18		
Unknown	8 602	22		
Sex				
Male	33 792	88		
Female	4 757	12		
Plant ^a^				
Plant 1	9 090	24		
Plant 2	17 087	44		
Plant 3	12 372	32		
Ever exposed to MWF				
Straight	20 352	53		
Soluble	31 795	82		
Synthetic	12 523	32		
Deceased by end of follow-up	20 565	53		
Years of follow-up			39	34, 47
Years at work ^b^			16.6	7.5, 27.3
Year of hire			1965	1952, 1973
Age at hire (years)			28	23, 36
Year of birth			1937	1922, 1948
Year of death among deceased			1996	1984, 2006
Age at death (years) among deceased			70	60, 79
Cumulative exposure to MWF ^c^ (mg/m^3^ y)				
Straight			0.66	0.21, 2.34
Soluble			4.41	1.74, 10.71
Synthetic			0.44	0.15, 1.56

a For individuals who worked at several plants, plant was taken to be the site where they accrued the most work record time.

b Among those with known date of leaving work.

c Summary statistics calculated for exposed individuals at end of follow-up only. Exposures were lagged 21 years.

### Standardized mortality ratios

SMR are presented for specific cancers and other major causes of death in table 2. The SMR for all causes of death combined was <1.0. This was driven by the low SMR for all heart disease (SMR=0.75) as well as nonmalignant respiratory diseases (SMR=0.84) and cerebrovascular disease (SMR=0.83). The SMR for all cancers was also <1.0. Although the majority of the SMR for specific cancers were <1.0, the SMR for some digestive and respiratory cancers was elevated including for esophageal (SMR=1.06), stomach (SMR=1.10), pancreatic (SMR=1.05), laryngeal (SMR=1.17) and lung (SMR=1.07) cancers. The SMR for lung cancer was the only one that was positive and statistically significant.

**Table 2:** Standardized mortality ratios (SMR) calculated for the GM-UAW cohort (1941–2015). NIOSH LTAS-extracted reference rates were used from 1940–2009 and CDC mortality data from 2010 onwards.

Cause of death	N	SMR	95% CI ^a^
All causes	20 565	0.91	0.89–0.92
All natural causes	18 857	0.89	0.88–0.91
All cancers	5 472	0.96	0.94–0.99
Esophageal cancer	184	1.06	0.92–1.23
Stomach cancer	192	1.10	0.95–1.27
Intestinal cancer	418	0.90	0.82–0.99
Rectal cancer	89	0.86	0.70–1.06
Bladder and urinary organ cancers	146	0.95	0.81–1.12
Bile duct, liver, and gallbladder cancers	162	0.88	0.76–1.03
Pancreatic cancer	315	1.05	0.94–1.17
Laryngeal cancer	74	1.17	0.93–1.47
Lung cancer	1891	1.07	1.02–1.12
Skin cancer	73	0.66	0.52–0.83
Prostate cancer	417	0.82	0.75–0.91
Brain and nervous system cancers	128	0.99	0.84–1.18
Leukemia	200	0.98	0.85–1.12
Breast cancer	76	0.79	0.63–0.99
All nonmalignant respiratory diseases	1 82	0.84	0.81–0.89
Chronic obstructive pulmonary disease	924	0.93	0.87–0.99
Cirrhosis and other chronic liver disease	379	0.90	0.81–1.00
All heart diseases	6 43	0.75	0.73–0.77
Ischemic heart disease	5056	0.89	0.87–0.92
Cerebrovascular disease	1080	0.83	0.78–0.88
All external causes	1671	1.03	0.98–1.08

a Variance estimates assume Poisson-distributed rates in the observed population.

### Proportional hazards models

**Figure 1:**
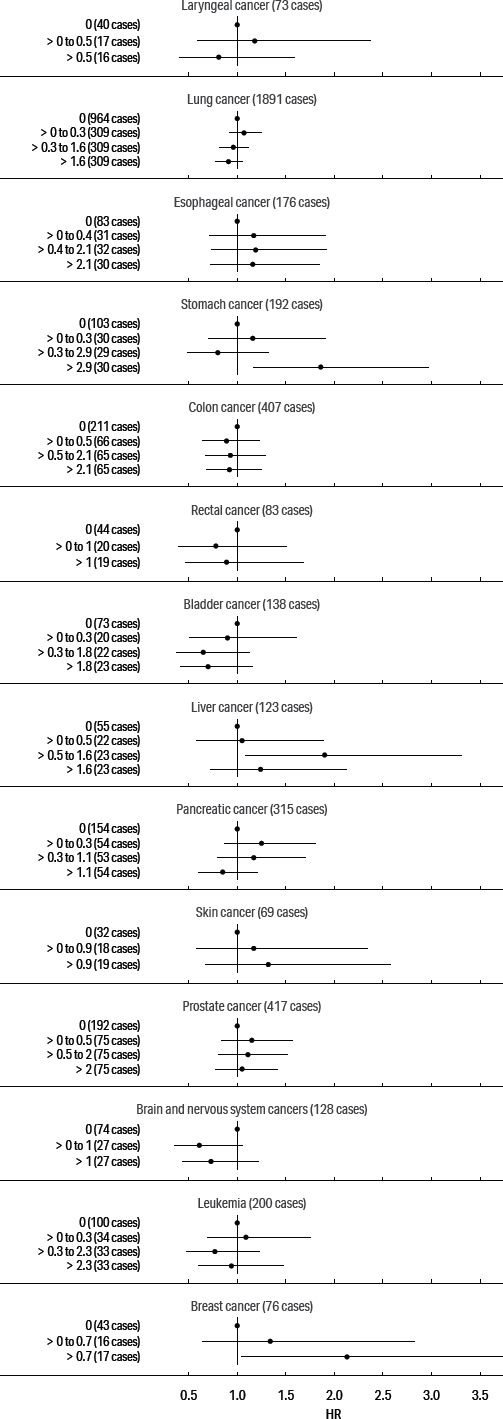
Adjusted hazard ratio estimates for cancers and cumulative exposure to straight metalworking fluids.

**Figure 2:**
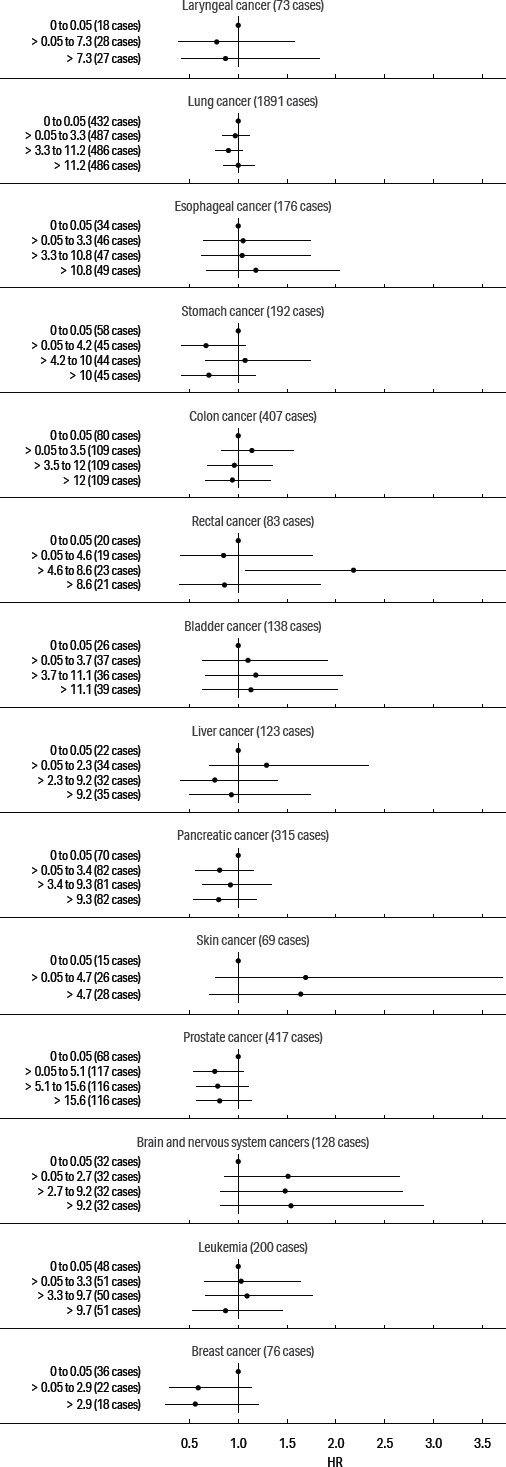
Adjusted hazard ratio estimates for cancers and cumulative exposure to soluble metalworking fluids.

**Figure 3:**
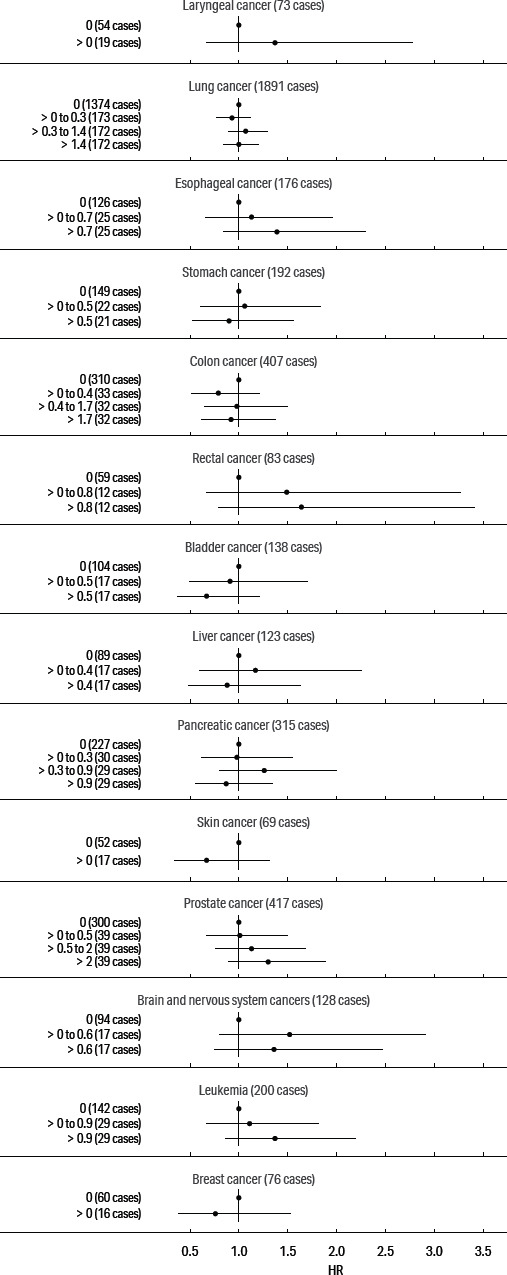
Adjusted hazard ratio estimates for cancers and cumulative exposure to synthetic metalworking fluids.

Our primary focus was on cancers of the digestive and respiratory systems: esophageal, stomach, rectal, lung, and larynx cancers; we also present models for pancreas, prostate, female breast, and skin cancers and for leukemia, based on previously elevated SMR. The adjusted HR for these cancers and cumulative exposure to straight, soluble, and synthetic MWF are presented in figures 1, 2, and 3, respectively. (See supplementary tables S2–4.)

The estimated exposure–response pattern for cumulative straight fluid was non-monotonic for all cancers except skin and breast cancer. In the highest exposure categories, skin cancer rose to a HR of 1.32 [95% confidence interval (CI) 0.67–2.58] and breast cancer to 2.13 (95% CI 1.04–4.39). Notably, the HR for stomach cancer was also highest in the highest category and rose to 1.86 (95% CI 1.17–2.97). The HR were mostly elevated for esophageal, liver, pancreatic, and prostate cancer in response to straight fluid exposure, but generally below the null for lung, colon, rectal, bladder, and brain cancers and leukemia.

The exposure–response patterns for exposure to cumulative soluble fluid were non-monotonic for all cancers. The HR for rectal cancer rose to 2.18 (95% CI 1.07–4.48) in the middle category. The HR were mostly elevated for esophageal, bladder, skin, and brain cancers in relation to soluble fluid, but generally below the null for laryngeal, lung, stomach, colon, liver, pancreatic, prostate, and breast cancers.

The exposure–response patterns for exposure to cumulative synthetic fluid were monotonic for esophageal, rectal, and prostate cancers and leukemia. The HR in the highest category was 1.39 (95% CI 0.84–2.30) for esophageal cancer, 1.64 (0.79–3.41) for rectal cancer, 1.30 (95% CI 0.89–1.89) for prostate cancer, and 1.37 (95% CI 0.86–2.19) for leukemia. In addition, the HR were generally elevated in response to cumulative synthetic fluid for laryngeal and brain cancers and below the null for colon, pancreatic, bladder, skin, and breast cancers.

Results did not change when we classified people with unknown race as either white or black in plant 1 (data not shown).

## Discussion

This updated report includes almost 5500 deaths from cancer, more than ten times the number of cancer deaths in our last summary report published almost 20 years ago. Most of the patterns reported here are consistent with that previous summary, as well as with results of cancer-specific papers published from this cohort during the interim and suggest that a malignancy-based OHSA standard for MWF would be appropriately health protective. For example, increasing straight fluid exposure was associated with increased risk of skin and female breast cancers. Interestingly, for the first time in this cohort, we report an increase in stomach cancer mortality with increasing straight fluid exposure, which was the original hypothesis motivating this cohort study. Although there are some suggestions of increased risk that we will explore in targeted analyses, many exposure–response results do not suggest any association. It is certainly possible that MWF simply do not predict many of these cause-specific cancers; however, there are also limitations which can lead to attenuation, including using mortality as a surrogate outcome for cancer diagnosis, a lack of data on potential confounders, such as smoking, and the healthy worker survivor effect.

Mortality may be a reasonable proxy for diagnosis for cancers with a poor 5-year survival rate, such as lung or pancreatic cancer. However, many cancers have become more highly treatable over the 75-year study period. Thus, cancer mortality is a measure that is bound to (i) be less sensitive for cancers with better 5-year survival and (ii) disproportionately include cancers that were diagnosed at later stages, were more aggressive or treated less effectively. Given the known social and racial disparities in medical care (31) and cancer survival (32), we assume that the cause-specific cancer deaths identified in this analysis are a non-random subset of all occurrences of cancer in this cohort. Mortality outcomes can also obfuscate a time-window or lagged analysis since date of death can be years after the first date of diagnosis. For these reasons, incidence is generally preferred to mortality as an outcome measure for cancer etiology studies.

Mortality does, however, offer some advantages as an outcome over incidence. The Michigan Cancer Registry started in 1985, and linkage can identify cancer incidence in the cohort, but limited to diagnoses in the state of Michigan that occurred after the initiation of the registry. This data structure can lead to increased potential for misclassified outcomes and survivor bias due to left truncation. Thus, although mortality may not be the best outcome for studying the increased risk of cancer from an occupational exposure, it does allow us to leverage the full cohort of almost 40 000 workers followed for up to 75 years.

There are known risk factors for many of the cancers presented in this paper that were not measured in this cohort, for example, *Helicobacter pylori* infection for stomach cancer, sun exposure for skin cancer, diet for rectal cancer, and parity for breast cancer. Clearly, not all risk factors need to be adjusted for, however, those that are also associated with exposure need to be. Given the lack of association between most of the cancers and MWF, we considered whether we were missing information on a ubiquitous risk factor that might be inversely associated with increased MWF exposure. That is, is there a risk factor for mortality from several cancers that is more likely to occur among the unexposed? In this cohort, assembly workers were classified as unexposed to each specific type of MWF and comprise a large portion of the reference group for all fluid types, but especially soluble fluid. If assembly workers were more likely to be exposed to other occupational chemicals, smoke cigarettes, or have less favorable socio-economic status than machine operators or machinists, our results could be globally attenuated due to confounding. Unfortunately, we are not able to test this theory since we do not have smoking or socio-economic data for members of our cohort.

Due to the quantitative exposure assessment of MWF, this UAW-GM cohort study has contributed substantially to our understanding of the health effects of MWF. However, any exposure assessment based on a job exposure matrix will result in some non-differential exposure misclassification which would likely result in attenuation of results. Additionally, the necessary use of a 21-year lag may also lead to attenuation, especially for cancers with shorter latency.

Our final area of concern is attenuation from the healthy worker effect (33). We present both SMR, using an external reference group, and Cox models, using an internal reference group. The SMR is known to suffer from the healthy worker hire effect because people who are hired into physically demanding jobs are healthier at baseline than the general population. Thus, SMR can mask a harmful effect of occupational exposures. Cox models avoid this well-known bias by using unexposed workers as the reference group. Even internal analyses can be attenuated from the healthy worker survivor effect, however, because workers who are the least susceptible to the ill effects of an occupational exposure stay at work the longest and accrue the most exposure. The use of a 21-year lagged exposure metric diminishes the problem, but does not account for any self-selection out of the work force that occurred prior to 21 years before cancer mortality. Of note, we avoided a portion of healthy worker survivor effect, known as left truncation bias (34), by only including workers who were hired after the start of follow up in 1941. However, eligibility into the study required three years of work prior to entering follow up. We expect that those who survived the first three years of work may be different from those that left earlier and therefore note that there is built-in left truncation bias by study design. Other than restricting to those hired after the start of follow up, we did not address the healthy worker survivor effect in this manuscript.

We report elevations in skin, breast and stomach cancer mortality from long term occupational exposure to MWF. Several excess cancer risks previously reported in this cohort have become closer to the null with extended follow-up. Before concluding that MWF exposures are not associated with other cancers, possible attenuation by the healthy worker survivor effect should be excluded. If leaving work is a time dependent confounder of future exposure and the outcome and caused by previous exposure, then the Cox model is not adequate (35). Despite the extensive exposure assessment, large sample size and long follow-up, causal inference methods such as g-methods (36) may also be necessary to avoid underestimation.

## Supplementary material

Supplementary material
