# Supplementary material for: Metalworking fluids and cancer mortality in a US autoworker cohort (1941–2015)
Source: Scand J Work Environ Health. 2020 May 14;46(5):525–32. doi: 10.5271/sjweh.3898 (PMC7737797; doi:10.5271/sjweh.3898)
Supplement: Supplementary material [file SJWEH-46-525-S001.pdf]

Metalworking fluids and cancer mortality in a US autoworker cohort (1941–2015)[1]

by Sadie Costello, PhD,[2] Kevin Chen, MPH, Sally Picciotto, PhD, Liza Lutzker, MPH, Ellen Eisen, ScD

1. Appendix
2. Correspondence to: Sadie Costello, PhD, Environmental Health Science, School of Public Health, University of California, Berkeley, 2121 Berkeley Way #5302, Berkeley, CA 94720-7360, USA. [E-mail: Sadie@berkeley.edu]

Table S1. International Classification of Diseases (ICD) codes used to define cases. Deaths prior to 1999 were defined with respect to the 9th revision. Deaths in or after 1999 were defined with respect to the 10th.

Cause of death	ICD 9	ICD 10
All causes	–	–
All natural causes	001–799	A00–R99, U00–Z99
All cancers	140–239	C00–C99, D00–D49
Esophageal cancer	150	C15
Stomach cancer	151	C16
Intestinal cancer	152–153	C17–C18
Rectal cancer	154	C19–C21
Kidney cancer	189.0, 189.2	C64–C66
Bladder and urinary organ cancers	188, 189.3–189.9	C67–C68
Bile duct, liver, and gallbladder cancers	155–156	C22–C24
Pancreatic cancer	157	C25
Laryngeal cancer	161	C32
Lung cancer	162	C33–C34
Skin cancer	172–173	C43–C44, C46.0, C46.9
Prostate cancer	185	C61
Brain and nervous system cancers	191–192	C47, C70–C72
Leukemia	204–208	C91.0–C91.3, C91.5–C91.9, C92–C95
Breast cancer	174–175	C50
All nonmalignant respiratory diseases	460–466, 470–478, 480–487, 490–495, 496–519	A48.1, J00–J01, J02.8–J02.9, J03.8–J03.9, J04–J06, J10–J18, J20–J22, J40–J46, J30–J33, J34.1–J34.8, J35–J39, J47, J60:J95, J98, R09.1
Chronic obstructive pulmonary disease	490–492, 496	J40–J44
Pneumonia	480–486	A48.1, J12–J18

Cirrhosis and other chronic liver disease	571	K70, K73–K74, K76.0
All heart diseases	390–398, 402, 404, 410–414, 420–429	I00–I09, I11–I13, I20–I22, I24–I25, I30–I38, I40, I42, I44–I52, I97.0–I97.1, I97.8–I97.9, R00.1, R00.8
Ischemic heart disease	410–414	I20–I22, I24–I25, I51.3, I51.6
Rheumatic heart disease	390–398	I00–I09
Cerebrovascular disease	430–438	G45.0–G45.2, G45.4–G45.9, I60–I69
All external causes	E800–E848, E850–E888, E929–E978, E980–E999	V00–V99, W00-W99, X00–X84, X85–X99, Y00–Y36, Y40–Y89

Table S2. Cox model estimates of the hazard ratio for selected cancer outcomes associated with exposure to straight metalworking fluids, controlling for other fluid types, calendar year, calendar year of hire, age, race, sex, and plant.

			Number of cases	HR	95% CI
Laryngeal cancer (73 cases)					
	0	mg/m^3·years	40	1.00	–
	> 0 to 0.5	mg/m^3·years	17	1.18	(0.59–2.37)
	> 0.5	mg/m^3·years	16	0.81	(0.41–1.59)
Lung cancer (1891 cases)					
	0	mg/m^3·years	964	1.00	–
	> 0 to 0.3	mg/m^3·years	309	1.07	(0.92–1.25)
	> 0.3 to 1.6	mg/m^3·years	309	0.96	(0.82–1.12)
	> 1.6	mg/m^3·years	309	0.91	(0.78–1.05)
Esophageal cancer (176 cases)					
	0	mg/m^3·years	83	1.00	–
	> 0 to 0.4	mg/m^3·years	31	1.17	(0.71–1.91)
	> 0.4 to 2.1	mg/m^3·years	32	1.19	(0.74–1.92)
	> 2.1	mg/m^3·years	30	1.16	(0.73–1.85)

Stomach cancer (192 cases)						
	0	mg/m³·years	103	1.00	–	
	> 0 to 0.3	mg/m³·years	30	1.16	(0.70–1.91)	
	> 0.3 to 2.9	mg/m³·years	29	0.80	(0.49–1.32)	
	> 2.9	mg/m³·years	30	1.86	(1.17–2.97)	*
Colon cancer (407 cases)						
	0	mg/m³·years	211	1.00	–	
	> 0 to 0.5	mg/m³·years	66	0.89	(0.64–1.23)	
	> 0.5 to 2.1	mg/m³·years	65	0.93	(0.67–1.29)	
	> 2.1	mg/m³·years	65	0.92	(0.68–1.25)	
Rectal cancer (83 cases)						
	0	mg/m³·years	44	1.00	–	
	> 0 to 1	mg/m³·years	20	0.78	(0.40–1.51)	
	> 1	mg/m³·years	19	0.89	(0.47–1.68)	
Bladder cancer (138 cases)						
	0	mg/m³·years	73	1.00	–	
	> 0 to 0.3	mg/m³·years	20	0.90	(0.51–1.61)	
	> 0.3 to 1.8	mg/m³·years	22	0.65	(0.37–1.13)	
	> 1.8	mg/m³·years	23	0.70	(0.42–1.16)	
Liver cancer (123 cases)						
	0	mg/m³·years	55	1.00	–	
	> 0 to 0.5	mg/m³·years	22	1.05	(0.58–1.89)	
	> 0.5 to 1.6	mg/m³·years	23	1.90	(1.09–3.31)	*
	> 1.6	mg/m³·years	23	1.24	(0.72–2.13)	
Pancreatic cancer (315 cases)						
	0	mg/m³·years	154	1.00	–	

	> 0 to 0.3	mg/m³·years	54	1.25	(0.87–1.81)	
	> 0.3 to 1.1	mg/m³·years	53	1.17	(0.80–1.70)	
	> 1.1	mg/m³·years	54	0.85	(0.60–1.21)	
Skin cancer (69 cases)						
	0	mg/m³·years	32	1.00	–	
	> 0 to 0.9	mg/m³·years	18	1.17	(0.58–2.34)	
	> 0.9	mg/m³·years	19	1.32	(0.67–2.58)	
Prostate cancer (417 cases)						
	0	mg/m³·years	192	1.00	–	
	> 0 to 0.5	mg/m³·years	75	1.15	(0.84–1.57)	
	> 0.5 to 2	mg/m³·years	75	1.11	(0.81–1.52)	
	> 2	mg/m³·years	75	1.05	(0.78–1.41)	
Brain and nervous system cancers (128 cases)						
	0	mg/m³·years	74	1.00	–	
	> 0 to 1	mg/m³·years	27	0.61	(0.35–1.05)	.
	> 1	mg/m³·years	27	0.73	(0.44–1.22)	
Leukemia (200 cases)						
	0	mg/m³·years	100	1.00	–	
	> 0 to 0.3	mg/m³·years	34	1.09	(0.69–1.75)	
	> 0.3 to 2.3	mg/m³·years	33	0.77	(0.48–1.23)	
	> 2.3	mg/m³·years	33	0.94	(0.60–1.48)	
Breast cancer (76 cases)						
	0	mg/m³·years	43	1.00	–	
	> 0 to 0.7	mg/m³·years	16	1.34	(0.64–2.82)	
	> 0.7	mg/m³·years	17	2.13	(1.04–4.39)	*

Table S3. Cox model estimates of the hazard ratio for selected cancer outcomes associated with exposure to soluble metalworking fluids, controlling for other fluid types, calendar year, calendar year of hire, age, race, sex, and plant.

			Number of cases	HR	95% CI
Laryngeal cancer (73 cases)					
	0 to 0.05	mg/m^3·years	18	1.00	–
	> 0.05 to 7.3	mg/m^3·years	28	0.78	(0.39–1.58)
	> 7.3	mg/m^3·years	27	0.87	(0.42–1.83)
Lung cancer (1891 cases)					
	0 to 0.05	mg/m^3·years	432	1.00	–
	> 0.05 to 3.3	mg/m^3·years	487	0.97	(0.84–1.12)
	> 3.3 to 11.2	mg/m^3·years	486	0.90	(0.77–1.04)
	> 11.2	mg/m^3·years	486	1.00	(0.85–1.17)
Esophageal cancer (176 cases)					
	0 to 0.05	mg/m^3·years	34	1.00	–
	> 0.05 to 3.3	mg/m^3·years	46	1.05	(0.64–1.74)
	> 3.3 to 10.8	mg/m^3·years	47	1.04	(0.62–1.74)
	> 10.8	mg/m^3·years	49	1.18	(0.68–2.04)
Stomach cancer (192 cases)					
	0 to 0.05	mg/m^3·years	58	1.00	–
	> 0.05 to 4.2	mg/m^3·years	45	0.67	(0.42–1.07)
	> 4.2 to 10	mg/m^3·years	44	1.07	(0.66–1.74)
	> 10	mg/m^3·years	45	0.70	(0.42–1.18)
Colon cancer (407 cases)					
	0 to 0.05	mg/m^3·years	80	1.00	–
	> 0.05 to 3.5	mg/m^3·years	109	1.14	(0.83–1.57)
	> 3.5 to 12	mg/m^3·years	109	0.96	(0.69–1.35)

	> 12	mg/m^3·years	109	0.94	(0.66–1.33)	
Rectal cancer (83 cases)						
	0 to 0.05	mg/m^3·years	20	1.00	–	
	> 0.05 to 4.6	mg/m^3·years	19	0.85	(0.41–1.76)	
	> 4.6 to 8.6	mg/m^3·years	23	2.18	(1.07–4.48)	*
	> 8.6	mg/m^3·years	21	0.86	(0.40–1.84)	
Bladder cancer (138 cases)						
	0 to 0.05	mg/m^3·years	26	1.00	–	
	> 0.05 to 3.7	mg/m^3·years	37	1.10	(0.63–1.91)	
	> 3.7 to 11.1	mg/m^3·years	36	1.18	(0.67–2.07)	
	> 11.1	mg/m^3·years	39	1.13	(0.63–2.02)	
Liver cancer (123 cases)						
	0 to 0.05	mg/m^3·years	22	1.00	–	
	> 0.05 to 2.3	mg/m^3·years	34	1.29	(0.71–2.33)	
	> 2.3 to 9.2	mg/m^3·years	32	0.76	(0.41–1.40)	
	> 9.2	mg/m^3·years	35	0.93	(0.50–1.74)	
Pancreatic cancer (315 cases)						
	0 to 0.05	mg/m^3·years	70	1.00	–	
	> 0.05 to 3.4	mg/m^3·years	82	0.81	(0.56–1.16)	
	> 3.4 to 9.3	mg/m^3·years	81	0.92	(0.63–1.34)	
	> 9.3	mg/m^3·years	82	0.80	(0.54–1.19)	
Skin cancer (69 cases)						
	0 to 0.05	mg/m^3·years	15	1.00	–	
	> 0.05 to 4.7	mg/m^3·years	26	1.69	(0.77–3.70)	
	> 4.7	mg/m^3·years	28	1.64	(0.71–3.80)	
Prostate cancer (417 cases)						

0 to 0.05	mg/m³·years	68	1.00	–
> 0.05 to 5.1	mg/m³·years	117	0.76	(0.54–1.05)
> 5.1 to 15.6	mg/m³·years	116	0.79	(0.57–1.11)
> 15.6	mg/m³·years	116	0.81	(0.57–1.14)
Brain and nervous system cancers (128 cases)				
0 to 0.05	mg/m³·years	32	1.00	–
> 0.05 to 2.7	mg/m³·years	32	1.51	(0.86–2.65)
> 2.7 to 9.2	mg/m³·years	32	1.48	(0.82–2.68)
> 9.2	mg/m3·years	32	1.54	(0.82–2.90)
Leukemia (200 cases)				
0 to 0.05	mg/m³·years	48	1.00	–
> 0.05 to 3.3	mg/m³·years	51	1.03	(0.65–1.64)
> 3.3 to 9.7	mg/m³·years	50	1.09	(0.67–1.76)
> 9.7	mg/m³·years	51	0.87	(0.53–1.45)
Breast cancer (76 cases)				
0 to 0.05	mg/m³·years	36	1.00	–
> 0.05 to 2.9	mg/m³·years	22	0.59	(0.30–1.14)
> 2.9	mg/m³·years	18	0.56	(0.26–1.21)

Table S4. Cox model estimates of the hazard ratio for selected cancer outcomes associated with exposure to synthetic metalworking fluids, controlling for other fluid types, calendar year, calendar year of hire, age, race, sex, and plant.

		Number of cases	HR	95% CI
Laryngeal cancer (73 cases)				
0	mg/m³·years	54	1.00	–
> 0	mg/m³·years	19	1.37	(0.67–2.78)
Lung cancer (1891 cases)				
0	mg/m³·years	1374	1.00	–

	> 0 to 0.3	mg/m^3·years	173	0.93	(0.77–1.12)
	> 0.3 to 1.4	mg/m^3·years	172	1.07	(0.89–1.29)
	> 1.4	mg/m^3·years	172	1.00	(0.84–1.20)
Esophageal cancer (176 cases)					
	0	mg/m^3·years	126	1.00	–
	> 0 to 0.7	mg/m^3·years	25	1.13	(0.66–1.96)
	> 0.7	mg/m^3·years	25	1.39	(0.84–2.30)
Stomach cancer (192 cases)					
	0	mg/m^3·years	149	1.00	–
	> 0 to 0.5	mg/m^3·years	22	1.06	(0.61–1.84)
	> 0.5	mg/m^3·years	21	0.90	(0.53–1.56)
Colon cancer (407 cases)					
	0	mg/m^3·years	310	1.00	–
	> 0 to 0.4	mg/m^3·years	33	0.79	(0.52–1.21)
	> 0.4 to 1.7	mg/m^3·years	32	0.98	(0.65–1.50)
	> 1.7	mg/m^3·years	32	0.92	(0.62–1.38)
Rectal cancer (83 cases)					
	0	mg/m^3·years	59	1.00	–
	> 0 to 0.8	mg/m^3·years	12	1.49	(0.67–3.27)
	> 0.8	mg/m^3·years	12	1.64	(0.79–3.41)
Bladder cancer (138 cases)					
	0	mg/m^3·years	104	1.00	–
	> 0 to 0.5	mg/m^3·years	17	0.91	(0.49–1.70)
	> 0.5	mg/m^3·years	17	0.67	(0.37–1.21)
Liver cancer (123 cases)					
	0	mg/m^3·years	89	1.00	–

	> 0 to 0.4	mg/m^3·years	17	1.17	(0.60–2.26)
	> 0.4	mg/m^3·years	17	0.88	(0.48–1.63)
Pancreatic cancer (315 cases)					
	0	mg/m^3·years	227	1.00	–
	> 0 to 0.3	mg/m^3·years	30	0.98	(0.62–1.55)
	> 0.3 to 0.9	mg/m^3·years	29	1.26	(0.80–2.00)
	> 0.9	mg/m^3·years	29	0.87	(0.56–1.34)
Skin cancer (69 cases)					
	0	mg/m^3·years	52	1.00	–
	> 0	mg/m^3·years	17	0.67	(0.34–1.31)
Prostate cancer (417 cases)					
	0	mg/m^3·years	300	1.00	–
	> 0 to 0.5	mg/m^3·years	39	1.01	(0.67–1.50)
	> 0.5 to 2	mg/m^3·years	39	1.13	(0.76–1.68)
	> 2	mg/m^3·years	39	1.30	(0.89–1.89)
Brain and nervous system cancers (128 cases)					
	0	mg/m^3·years	94	1.00	–
	> 0 to 0.6	mg/m^3·years	17	1.52	(0.80–2.91)
	> 0.6	mg/m^3·years	17	1.36	(0.75–2.47)
Leukemia (200 cases)					
	0	mg/m^3·years	142	1.00	–
	> 0 to 0.9	mg/m^3·years	29	1.11	(0.67–1.82)
	> 0.9	mg/m^3·years	29	1.37	(0.86–2.19)
Breast cancer (76 cases)					
	0	mg/m^3·years	60	1.00	–
	> 0	mg/m^3·years	16	0.76	(0.38–1.53)
